# Characterization of the genetic diversity of *Mycobacterium tuberculosis *in São Paulo city, Brazil

**DOI:** 10.1186/1756-0500-4-269

**Published:** 2011-07-29

**Authors:** Natália H Mendes, Fernando AF Melo, Adolfo CB Santos, José RC Pandolfi, Elisabete A Almeida, Rosilene F Cardoso, Henri Berghs, Suzana David, Faber K Johansen, Lívia G Espanha, Sergio RA Leite, Clarice QF Leite

**Affiliations:** 1 Laboratory of Micobacteriology, Faculty of Pharmacy, São Paulo State University at Araraquara, UNESP. Araraquara-Jaú Road Km 01, Araraquara, SP, 14801-902, Brazil; 2 Clemente Ferreira Institute. 717 Consolação St, São Paulo, SP, 01301-000, Brazil; 3 State University of Maringá. 5790 Colombo Ave, Maringá, PR, 87020-900, Brazil; 4 Fairport Ltda. 293 Jacarandá St, São Paulo, SP, 04926-160, Brazil; 5 National Institute of Heath Dr. Ricardo Jorge. Padre Cruz Ave, Lisbon, 1649-016, Portugal; 6 Institute of Chemistry, São Paulo State University at Araraquara, UNESP, 55 Prof. Francisco Degni St, Araraquara, SP, 14800-060, Brazil

## Abstract

**Background:**

Tuberculosis is a major health problem in São Paulo, Brazil, which is the most populous and one of the most cosmopolitan cities in South America. To characterize the genetic diversity of *Mycobacterium tuberculosis *in the population of this city, the genotyping techniques of spoligotyping and MIRU were applied to 93 isolates collected in two consecutive years from 93 different tuberculosis patients residing in São Paulo city and attending the Clemente Ferreira Institute (the reference clinic for the treatment of tuberculosis).

**Findings:**

Spoligotyping generated 53 different spoligotype patterns. Fifty-one isolates (54.8%) were grouped into 13 spoligotyping clusters. Seventy- two strains (77.4%) showed spoligotypes described in the international databases (SpolDB4, SITVIT), and 21 (22.6%) showed unidentified patterns. The most frequent spoligotype families were Latin American Mediterranean (LAM) (26 isolates), followed by the T family (24 isolates) and Haarlem (H) (11 isolates), which together accounted for 65.4% of all the isolates. These three families represent the major genotypes found in Africa, Central America, South America and Europe. Six Spoligo-International-types (designated SITs by the database) comprised 51.8% (37/72) of all the identified spoligotypes (SIT53, SIT50, SIT42, SIT60, SIT17 and SIT1). Other SITs found in this study indicated the great genetic diversity of *M. tuberculosis*, reflecting the remarkable ethnic diversity of São Paulo city inhabitants. The MIRU technique was more discriminatory and did not identify any genetic clusters with 100% similarity among the 93 isolates. The allelic analysis showed that MIRU loci 26, 40, 23 and 10 were the most discriminatory. When MIRU and spoligotyping techniques were combined, all isolates grouped in the 13 spoligotyping clusters were separated.

**Conclusions:**

Our data indicated the genomic stability of over 50% of spoligotypes identified in São Paulo and the great genetic diversity of *M. tuberculosis *isolates in the remaining SITs, reflecting the large ethnic mix of the São Paulo city inhabitants. The results also indicated that in this city, *M. tuberculosis *isolates acquired drug resistance independently of genotype and that resistance was more dependent on the selective pressure of treatment failure and the environmental circumstances of patients.

## Findings

Tuberculosis (TB) is an infectious disease characterized by high morbidity and mortality in developing countries and in urban areas of developed countries [1]. Tuberculosis is a major health problem in São Paulo city with an incidence of 65.2 cases/100,000 inhabitants [2]. The city is considered the most cosmopolitan in South America, owing to its population density of more than 7,000 inhabitants per square kilometre and the very large number of registered immigrants [3].

To characterize the genetic diversity of the *Mycobacterium tuberculosis *strains responsible for TB in the population of São Paulo city, the genotyping techniques of spoligotyping and MIRU were applied to 93 strains isolated from clinical samples taken over two consecutive years from 93 different TB patients residing in this city, who were attending the Clemente Ferreira Institute for treatment.

Spoligotyping has been widely used in the molecular epidemiology of TB, to investigate the population structure of *M. tuberculosis*, focusing on the identification of genotypic lineages and spoligotype families and their geographic distribution [4]. Furthermore, molecular techniques such as MIRU (Mycobacterial Interspersed Repetitive Units) can be used to cluster *M. tuberculosis *strains from patients with epidemiological links, identify chains of transmission and discriminate isolates at the clonal level [5]. Spoligotyping is based on DNA polymorphisms within the direct repeat (DR) locus of *M. tuberculosis*, assaying the presence or absence of a set of target spacer sequences between the DRs. The binary pattern of spacers between the conserved DRs in the region is used to differentiate strains of *M. tuberculosis *[6]. MIRU genotyping is a highly reproducible and fast system, generating genotypes based on a detailed study of 12 loci of the *M. tuberculosis *genome, with structurally similar or, in some loci, identical Variable Number of Tandem Repeat (VNTR) mini-satellites [7]. MIRU genotyping applied as a first line molecular typing method, in combination with spoligotyping, has proved to be adequate for strain discrimination in most cases, including large-scale studies [8].

This paper is a report on the genetic diversity of *M. tuberculosis *isolates collected over 2 years from patients in São Paulo city, in southeast Brazil, who attended the Clemente Ferreira Institute.

## Methods

### Clinical isolates

From August 2006 to July 2008 a total of 93 *M. tuberculosis *isolates were obtained from sputum specimens of patients with pulmonary TB admitted to the Clemente Ferreira Institute, a health reference center for TB treatment in São Paulo city. All isolates were cultured on Löwenstein-Jensen (LJ) slants at 37°C, identified as *M. tuberculosis *by phenotypic [9] and PCR-IS6110 [10] methods and tested for drug susceptibility by the Becton-Dickinson MGIT 960 system [11]. The study had ethical approval from the Research Ethics Commitee of the School of Pharmaceutical Sciences, São Paulo State University at Araraquara (reference 01/2006).

### Spoligotyping

Briefly, *M. tuberculosis *cells from LJ slant cultures were lysed by freezing and boiling three times and the DNA (in solution) was purified by addition of phenol-chloroform-isoamyl alcohol (25:24:1, v/v), followed by chloroform-isoamyl alcohol (24:1, v/v) and then precipitated with ice-cold ethanol. The purified DNA pellet was resuspended in Tris-EDTA, pH 8.0 (TE buffer), and stored at -20°C until used [4].

The extracted DNA was subjected to PCR, to amplify the DR region, using 1 μL of mycobacterial DNA (10 ng) in 24 μL of a reaction mixture containing 0.4 μM of each primer, DRa 5'-GGTTTTGGGTCTGACGAC-3' (biotinylated 5' end) and DRb 5'-CCGAGAGGGGACGGAAAC-3' (both from Bioneer Company, South Korea), and the PCR Master Mix (Fermentas, USA). PCR products were hybridized with a set of 43 spacer oligonucleotides covalently linked to the spoligo-membrane (Pall Biosystems, Portsmouth, UK), according to the manufacturer's instructions. Bound fragments were incubated with streptavidin-peroxidase conjugate (Boehringer, Ingelheim, Germany) and then detected by chemiluminescence and assessed by an enhanced chemiluminescence system (ECL detection kit, Amersham, Buckinghamshire, UK). The autoradiograms were developed after 30 minutes of exposure, using standard photochemical products [4].

The term Shared Type (ST) designates spoligotypes common to more than one isolate. These have been assigned "Spoligo-International-Type" (SIT) numbers at the international database SITVIT, allowing online query of the current version in the published SpolDB4 database [12] and at http://www.pasteur-guadeloupe.fr:8081/SITVITDemo/[13]. The SITs identified in this study were classified into spoligotype families and subfamilies with the help of the SpolDB4 database. For spoligotype patterns not previously reported in SpolDB4, the 'Spotclust' database was used [14]. This model takes into account knowledge of the evolution of the DR region and assigns spoligotype patterns to families and subfamilies, by means of a computer algorithm based on studies of the previous database SpolDB3 http://cgi2.cs.rpi.edu/~bennek/SPOTCLUST.html[15].

### MIRU-VNTR

The mycobacterial DNA was extracted by thermolysis [16]. For genotyping, the set of VNTRs, consisting of 12 MIRUs (MIRU *loci *2,4,10,16,20,23,24,26,27,31,39,40), was investigated as described in the literature [7,17]. The PCR amplicon was subjected to electrophoresis in 2.0% w/v agarose gel (Invitrogen Life Technologies, São Paulo, Brazil). DNA ladders of 50 and 100 bp (Invitrogen Life Technologies, São Paulo, Brazil) were used as molecular markers. The gels were stained with ethidium bromide and visualized under ultraviolet light and photodocumented with Alpha-imager 2200 (Alpha Innotech Corporation, San Leandro, CA, USA). The size of the PCR fragment for each locus was estimated by visual comparison with the molecular markers and the MIRU allele score was determined from the size as described [7,16]. The results from each of the 12 loci were combined to create a 12-digit allelic profile.

### Construction of dendrograms

The spoligotyping data, the MIRU data and a combination of both data sets were analysed with the software program BioNumerics 4.5 (Applied Maths^®^, Sint Martens Latem, Belgium), using Euclidean Distance to calculate the similarity matrix, and a dendrogram based on this matrix was constructed by the UPGMA method.

## Results

Of the 93 patients affected by tuberculosis, 56 (60.2%) were males and 37 (39.8%) females.

The drug resistance profiles of the 93 clinical isolates were as follows: 58.1% showed resistance to isoniazid (INH), 50.5% to rifampicin (RIF), 26.9% to ethambutol (EMB) and 34.4% to streptomycin (ST). Isolates simultaneously resistant to INH and RIF represented 49.5% (46) of the total number of isolates, whereas 25.8% (24) were resistant to INH, RIF and EMB and 17.2% (16) to INH, RIF, EMB and ST.

Colony morphology, biochemical characteristics and the presence of a 245 bp spoligotyping amplification product confirmed (in all 93 clinical isolates) the etiological agent *M. tuberculosis*.

Spoligotyping revealed that, of the 93 isolates, 72 (77.4%) showed spoligotypes described in the international database (SpolDB4) consisting of 36 different patterns, belonging to six families and 15 subfamilies (T1, T1 (T4-CE1), T2, T2-T3, T3, H1, H3, LAM1, LAM2, LAM3, LAM4, LAM5, LAM6, LAM9, X3, Beijing, S) and undesignated (U and U likely H) (Table 1).

**Table 1:** Spoligotypes identified in SpolDB4 by a SIT number^a^

SIT^a^	Spoligotype	Label^b^	No. of Isolates^c^
1	000000000003771	Beijing	4
47	777777774020771	H1	2
50	777777777720771	H3	8
467	000000000020771	H3	1
20	677777607760771	LAM 01	1
17	677737607760771	LAM 02	4
826	677737207760771	LAM 02	1
33	776177607760771	LAM 03	1
130	776177607760731	LAM 03	1
60	777777607760731	LAM 04	4
828	377777607760731	LAM 04	2
1895	777777607460731	LAM 04	1
93	777737607760771	LAM 05	1
64	777777607560771	LAM 06	1
42	777777607760771	LAM 09	7
1154	777777607760751	LAM 09	1
1277	777777207760771	LAM 09	1
34	776377777760771	S	1
53	777777777760771	T1	10
86	777777737760771	T1	1
102	777703777760771	T1	1
244	777777777760601	T1	1
453	777777677560771	T1	1
1166	777377777760771	T1	1
1214	777617777760771	T1	1
1475	777357777760771	T1	1
1905	777777777460771	T1	2
65	777777777760471	T1 (T4-CE1 )	1
175	777777677760731	T2	1
73	777737777760731	T2-T3	1
157	777737777760471	T3	2
397	777777600000771	U	2
534	777777607400000	U	1
560	777000000000371	U	1
46	777777770000000	U (likely H)	1
92	700076777760771	X3	1

^a^Spoligo International Type (SIT), International spoligotype database SpolDB4 www.pasteur-guadeloupe.fr:8081/SITVITDemo/; ^b^Label for spoligotype families as designated in the international spoligotype database SpolDB4; ^C^Number of isolates in this study.

**Figure 1:**
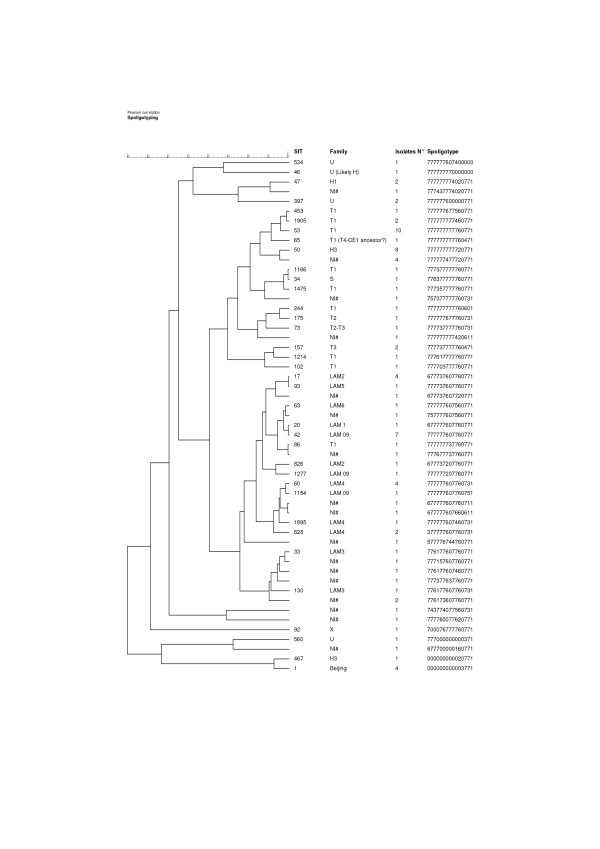
**Dendrogram produced with the results obtained by spoligotyping**.

Among the 72 isolates with spoligotypes described in SpolDB4, the Latin American-Mediterranean (LAM) and T families were the main spoligotype families, with 26 (36.1%) and 24 (33.3%) isolates respectively, followed by H, with 11 (15.3%) isolates. Thus, the LAM and T spoligotype families together totalled 69.4% of the all isolates. The Undesignated group (U) comprised 6.9% of isolates and the S and X families were each represented by a single isolate (Figure 1).

Twenty-one isolates (22.6%), representing 17 spoligotype patterns, could not be classified into SITs and families. These unidentified spoligotypes were analyzed by Spotclust, a program enabling the percent similarity of these patterns with existing subfamilies to be determined (Table 2).

**Table 2:** Spoligotypes analyzed by Spotclust.

No. of Isolates	Spoligotype	% Similarity/Subfamilies
4	777777477720771	11% T1 and 39% H3
1	776177607460771	99% LAM9
2	776173607760771	99% LAM3
1	677777607760711	33% LAM1
1	743774077560731	98% H37Rv
1	677777607660611	66% LAM1 and 34% LAM9
1	757777607560771	99% LAM9
1	777777777420611	66% H3 and 25% T1
1	777377637760771	99% T1
1	757377777760731	99% T1
1	677700000160771	99% T4
1	577776744760771	65% X1 and 34% T1
1	677737607720771	81% LAM2 and 10% LAM1
1	777677737760771	99% T1
1	777760077620771	96% H37Rv
1	777437774020771	99% H1
1	777157607760771	99% LAM9

The spoligotype dendrogram constructed with BioNumerics 4.5, presented in Figure 1, grouped 51 (54.8%) of the isolates into 13 clonal groups (two of them composed of unidentified clustered strains) with 100% similarity. Six groups consisted of two isolates each, four groups of four isolates each, one of seven isolates, one of eight isolates and one of ten isolates. Regarding the two clusters of unidentified spoligotype patterns, the dendrogram indicated that the spoligotype pattern with four clustered isolates showed 93% similarity with SIT 50 (subfamily H3), differing only by the absence of spacers 20 and 21. The other spoligotype pattern, with two clustered isolates, showed 87% similarity with SIT 130 (subfamily LAM3), differing in just two spacers (absence of spacer 16 and presence of spacer 39).

Genotyping by the MIRU technique generated 93 distinct genotypes and no formation of genetic groups with 100% similarity. The technique also revealed that the MIRU loci 10, 23, 26 and 40 were the most discriminatory. Loci 26 and 40 were the most polymorphic, with eight alleles, followed by loci 23 and 10 with seven alleles each. In all the strains tested, locus 24 showed 0 or 1 allele. Amplification was unsuccessful for locus 23 in one isolate and locus 40 in another, even after five repeats.

The dendrogram resulting from the combined analysis of spoligotyping and MIRU data, performed with the Bionumerics 4.5 program, showed no clonal group, thus generating 93 independent genotypes (data not shown).

Table 3 shows the isolates clustered by spoligotyping and their respective geographic distribution, MIRU and drug resistance profiles against isoniazid (INH), rifampicin (RIF), streptomycin (ST) and ethambutol (EMB).

**Table 3:** Summarized results of all assays

No. of Isolates	SIT	Label	Geographic distribution	MIRU Locus alleles	Drug resistance profile
4	1	Beijing	Ubiquitous common	223126152331	INH + RIF

				223321172412	INH + RIF
				223425173423	ST
				223325183503	INH + RIF + ST

2	397	U	ARG, BRA, ITA, USA, VEN	023224062103	INH + RIF + ST + EMB
				123204052113	INH + ST

2	47	H1	Ubiquitous common	225212052314	INH + RIF + EMB
				225313153314	S^b^

2		NI^a^		223115052313	INH + RIF
				224226153303	INH + RIF + EMB

4	60	LAM04	Ubiquitous common	124325132324	INH + ST
				023225132214	INH + RIF
				124326033304	INH + RIF + EMB
				224127131221	INH + RIF + ST + EMB

2	828	LAM04	BRA, GNB	225226143301	INH
				133223142322	INH + RIF + ST

7	42	LAM09	Ubiquitous common	123016052211	S
				124014041311	S
				124224042111	S
				123100141311	INH + RIF + ST + EMB
				224126041311	INH + RIF
				234126132311	INH
				224225132221	ST

4	17	LAM02	Ubiquitous common	223114162211	S
				224325143211	S
				224126153301	INH + RIF + ST + EMB
				224225132221	INH + RIF + ST + EMB

10	53	T1	Ubiquitous common	123225152322	INH + ST
				222014022204	S
				222215032215	S
				224225112121	INH + RIF + ST
				212223112213	INH + RIF + ST
				223325163214	S
				224225143301	INH + RIF + ST
				223126142301	INH + RIF + EMB
				204324113301	INH + RIF + EMB
				223024122328	S

2	1905	T1	ARG, BRA	124324042324	INH + RIF + EMB
				223113152314	RIF

2	157	T3	DEU, ITA, PRT	124323042212	S
				024215032114	INH

4		NI^a^		125202052212	INH + RIF + EMB
				124301152314	INH + RIF + ST
				22422?152322	-
				225324152326	INH + RIF + ST + EMB

8	50	H3	Ubiquitous common	225302152213	INH
				225214152313	INH + RIF + ST + EMB
				223202152313	S
				127213052312	INH + RIF + ST + EMB
				22331215232?	INH + RIF + ST + EMB
				225313153313	S^b^
				226324152324	INH + RIF + ST + EMB
				226223152224	INH + RIF + ST + EMB

## Discussion

The evaluation of epidemiological data from 93 patients revealed that 60.2% were male and 39.8% were female. Moreover, 46 (49.5%) of the *M. tuberculosis *clinical isolates were multi-drug resistant (MDR). In the same health center, it was found that, of 182 patients, 112 (61.5%) were male and that all of them presented MDR tuberculosis [18].

The main spoligotype families in this study, LAM and T (representing 69.4% of the isolates), were also found in the Hospital Fernando Fonseca in Lisbon, Portugal, where the predominance of the LAM family was noted in 51% of the isolates [19]. The preponderance of these *M. tuberculosis *families in São Paulo city and Lisbon is probably due to the close relationship between Brazil and Portugal, especially between the two cities. In Brazil, studies carried out in Rio Grande do Sul [20] and in Maringa (PR) [21] corroborate the prevalence of these families. The families LAM and T and also Haarlem are most frequently found in Africa, South America and Central Europe [12].

The other families found in isolates from São Paulo were the Beijing (four isolates), S and X, with one isolate each. The presence of the Beijing family (SIT1), representing 5.6% of all the identified SITs, may be due to the immigration of Asians to São Paulo city. In Portugal, the Beijing spoligotypes are less frequent (3%) [19]. Within the subfamily T1, we can highlight two isolates of a rare genotype SIT1905. Only two isolates of this genotype are cited in SpolDB4 (one in Brazil and the other in Argentina).

Analysing the geographic distribution of the spoligotypes, it appears that SIT1895 is a localized genotype described only in Brazil and that SIT 828 is found only in Brazil and Guinea Bissau. Fourteen spoligotypes (SITs: 33, 93, 157, 1154, 86, 102, 453, 467, 1166, 1214, 1475, 34, 534 and 560) not previously reported in Brazil were also observed. SIT 33 is a spoligotype identified in South Africa, but the presence of this pattern in São Paulo city may not be surprising. The north-eastern and south-eastern regions of Brazil have a strong African influence because of the slave trade in the past and, after its abolition, immigration from that continent. SIT93 is a spoligotype found in Venezuela and SIT 1154 in Haiti and the presence of these SITs in the city of São Paulo may be related to contact with those countries. The spoligotypes with SITs 86, 102, 453, 1166, 1214 and 1475 of the T1 subfamily, SIT 157 of the T3 as well as SIT 34 of the S family, originate in countries that have close immigration links to Brazil. In the mid 19th century, the southeast region (especially São Paulo) was the destination of many European immigrants, including Portuguese, Italians and Spaniards, in addition to immigrants from the Middle East, such as Syrians and Lebanese.

Analyzing the stability of the genotypes, our study indicated that six SITs (17, 60, 42, 53, 50 and 1) comprised 51.8% of all the isolates whose genotypes were identified in SpolDB4, indicating these as the major genotypes responsible for tuberculosis in São Paulo. In southern Brazil, it was observed that about half of the isolates belonged to seven spoligotypes (SITs 17, 20, 33, 42, 50, 53 and 65) [22]. Eight SITs responsible for 50% of the tuberculosis cases were also found in Lisbon, Portugal [19]. In Honduras, the five predominant spoligotypes were SIT33,42,67,53 and 376 [23]. The prevalence of 13.8%, 11.1% and 9.7% respectively of SITs 53, 50 and 42 was to be expected since these SITs are distributed worldwide [6]. SIT 53 is the second most frequently isolated type in SpolDB4, suggesting that this strain is easily transmitted and adaptable [24]. SITs 17 and 60 are also widely distributed around the world, although predominantly in Latin America [6,25]. In Rio Grande do Sul (south Brazil), the two patterns most frequently found were SIT50 and SIT53 [22]. Despite the fact that six SITs represent more than 50% of all spoligotypes identified in São Paulo, the other SITs found show the great genetic diversity of *M. tuberculosis *isolates, possibly reflecting the large ethnic mix of the São Paulo city inhabitants. Our data indicate that spoligotyping overestimated cluster formation, in agreement with the data in a previously published study [22] that investigated the genetic diversity of *M. tuberculosis *in southern Brazil, a region with a high incidence of tuberculosis.

The MIRU technique generated 93 independent genotypes and did not create any groups with 100% similarity, corroborating another study [20] in which it was shown that spoligotyping had a lower discriminatory power than MIRU. VNTR (minisatellites) are free of selective or functional pressure, so that MIRU profiles can be stable for up to 18 months of consecutive isolation [16]. Despite the two-year follow-up and study of 93 strains, our data can only show a tendency, owing to the small sample size in comparison with the high tuberculosis incidence in São Paulo. However, when applied to *M. tuberculosis *isolates from a rural population in Kanpur, South Asia, MIRU also failed to reveal genetic group formation [26]. The authors concluded that even in small populations, *M. tuberculosis *isolates may present high genetic diversity.

There is a hierarchy among the twelve polymorphic MIRU loci and it has been shown that loci 10, 23, 26, 31 and 40 generate the highest allelic diversity [6]. In agreement with that result, in our study loci 10, 23, 26, and 40 were the most discriminatory. However, locus 31 did not show high variation (ranging from alleles 1 to 5). In another study [20], loci 10, 23, 26 and 40 also showed the greatest discriminatory power.

MIRU locus 24 enables the classification of spoligotyping families into two groups (A and B). Group B has 0 or 1 allele at locus 24 and consists of families of more recent evolution. In our study, all isolates showed 0 or 1 allele at locus 24 and none was found to belong group A, with ancestral families (Bovis, AFRI or EAI) [27]. The isolates found in São Paulo thus belong to families that have evolved more recently and that are apparently more prevalent around the world.

The combination of the two techniques, spoligotyping and MIRU, separated all isolates of the 13 spoligotyping clusters, generating 93 different genotypes. Despite the small number of isolates in each of the spoligotyping clusters, certain MIRU loci were more discriminatory in differentiating SITs and subfamilies. It was observed that MIRU 40 was the most important in the discrimination of LAM, T and X families, and MIRU 26 and MIRU 23 in that of Beijing and Haarlem, respectively. Looking specifically at the 13 SITs clustered by spoligotyping (Table 3), our results agree with the literature with respect to T1 (locus 40) and Beijing (locus 26 for SIT1). However, for SITs 60 and 828 (LAM4), 42 (LAM9) and 17 (LAM2), locus 23 was the most promising, whereas for SIT50 from the Haarlem family, the most important was locus 10 [27].

From Table 3, we can also see that all isolates with the SIT1 pattern (Beijing family) were MDR strains, except for one isolate resistant only to streptomycin. The Beijing strains may be genetically more invasive and adapted to acquire drug resistance [28]. However, this study found that all the families identified comprised both MDR and non-MDR isolates. In Brazil, the characteristic of MDR in *M. tuberculosis *is mostly "acquired" as a result of treatment failure, due to irregularity in the taking of medication, neglect and incorrect prescriptions [29]. Our data corroborated these findings, indicating that in São Paulo city *M. tuberculosis *clinical isolates have acquired resistance to INH and RIF independently of their genotypes. MDR strains may have been generated by the selective pressure due to problems in adherence to treatment and environmental circumstances (high population density and ethnic diversity) of the inhabitants of São Paulo city.

## Competing interests

The authors declare that they have no competing interests.

## Authors' contributions

NM carried out molecular genetic studies, comprising spoligotyping, and drafted the manuscript. FM carried out the sampling of *Mycobacterium tuberculosis *and sent us the isolates. AS carried out the molecular techniques spoligotyping and MIRU-VNTR. JP, LE and FJ carried out the MIRU-VNTR technique. EA carried out the classic epidemiological analysis and SL the resistance profile of the samples. RF carried out the spoligotyping technique and helped in drafting this manuscript. HB helped in a construction of the dendrogram. SD carried out a draft of this manuscript. CL drafted the manuscript and is advisor and head of the laboratory.

All authors read and approved the final manuscript
